# Supplementary figures and images for: Matrix stiffness induces midnolin-dependent lamin B1 degradation to control myoblast differentiation
Source: EMBO Rep. 2026 Mar 31;27(9):2297–318. doi: 10.1038/s44319-026-00753-0 (PMC13172543; doi:10.1038/s44319-026-00753-0)

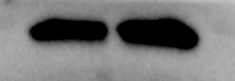

Supplement: Supplementary file 5 — Source data Fig. 3 [file 44319_2026_753_MOESM5_ESM.zip › Figure 3/3D/GAPDH.png]

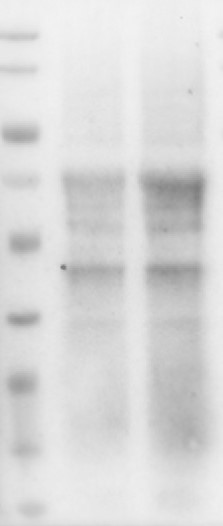

Supplement: Supplementary file 5 — Source data Fig. 3 [file 44319_2026_753_MOESM5_ESM.zip › Figure 3/3D/Puromycin.png]

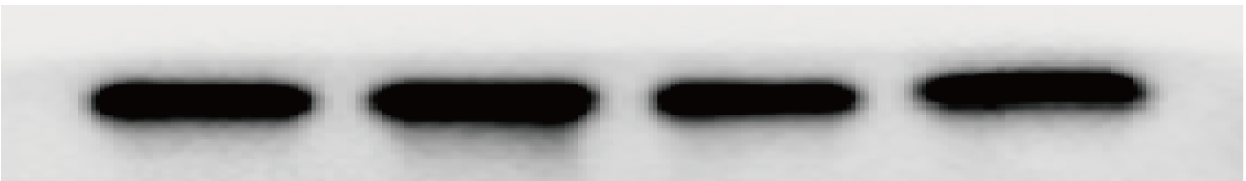

Supplement: Supplementary file 5 — Source data Fig. 3 [file 44319_2026_753_MOESM5_ESM.zip › Figure 3/3F/GAPDH.png]

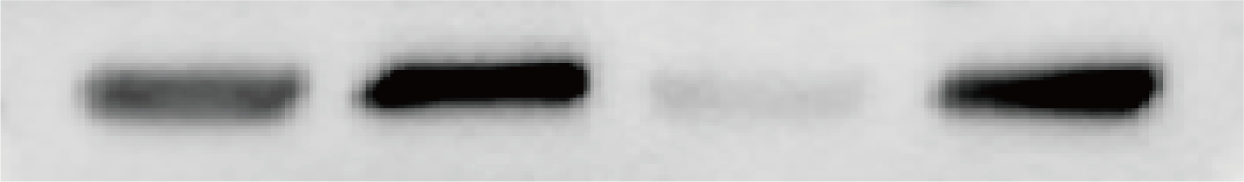

Supplement: Supplementary file 5 — Source data Fig. 3 [file 44319_2026_753_MOESM5_ESM.zip › Figure 3/3F/LaminB1.png]

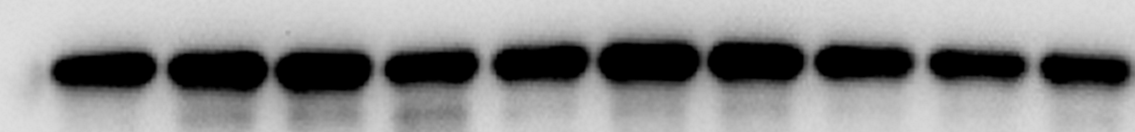

Supplement: Supplementary file 5 — Source data Fig. 3 [file 44319_2026_753_MOESM5_ESM.zip › Figure 3/3H/GAPDH.TIF]

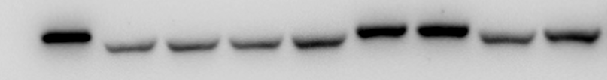

Supplement: Supplementary file 5 — Source data Fig. 3 [file 44319_2026_753_MOESM5_ESM.zip › Figure 3/3H/LaminB1.tif]

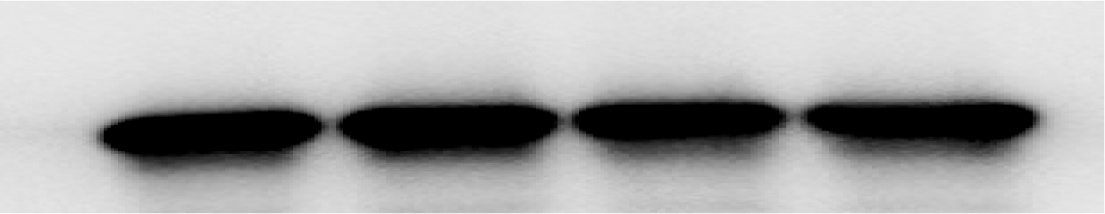

Supplement: Supplementary file 5 — Source data Fig. 3 [file 44319_2026_753_MOESM5_ESM.zip › Figure 3/3K/GAPDH.png]

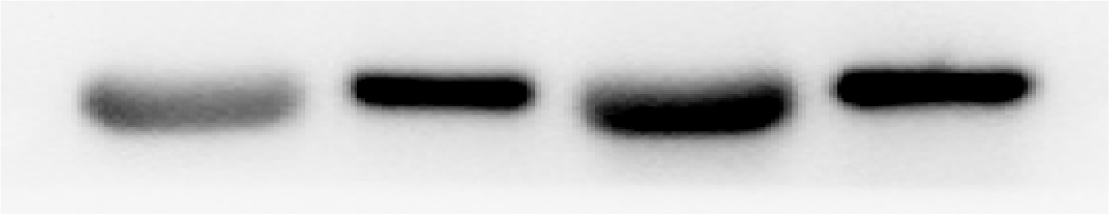

Supplement: Supplementary file 5 — Source data Fig. 3 [file 44319_2026_753_MOESM5_ESM.zip › Figure 3/3K/laminB1.png]

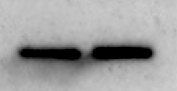

Supplement: Supplementary file 5 — Source data Fig. 3 [file 44319_2026_753_MOESM5_ESM.zip › Figure 3/3M/GAPDH.png]

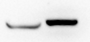

Supplement: Supplementary file 5 — Source data Fig. 3 [file 44319_2026_753_MOESM5_ESM.zip › Figure 3/3M/Lamin B1.png]

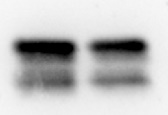

Supplement: Supplementary file 5 — Source data Fig. 3 [file 44319_2026_753_MOESM5_ESM.zip › Figure 3/3M/LC3.png]

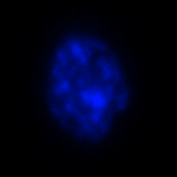

Supplement: Supplementary file 5 — Source data Fig. 3 [file 44319_2026_753_MOESM5_ESM.zip › Figure 3/3P/3P_Hoechst_BafA1.tiff]

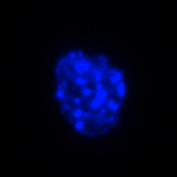

Supplement: Supplementary file 5 — Source data Fig. 3 [file 44319_2026_753_MOESM5_ESM.zip › Figure 3/3P/3P_Hoechst_DMSO.tiff]

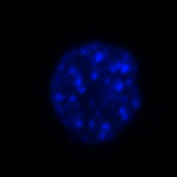

Supplement: Supplementary file 5 — Source data Fig. 3 [file 44319_2026_753_MOESM5_ESM.zip › Figure 3/3P/3P_Hoechst_MG132.tiff]

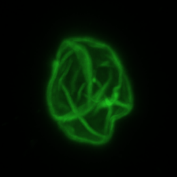

Supplement: Supplementary file 5 — Source data Fig. 3 [file 44319_2026_753_MOESM5_ESM.zip › Figure 3/3P/3P_LaminB1_BafA1.tiff]

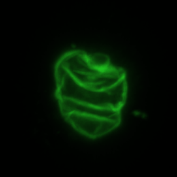

Supplement: Supplementary file 5 — Source data Fig. 3 [file 44319_2026_753_MOESM5_ESM.zip › Figure 3/3P/3P_LaminB1_DMSO.tiff]

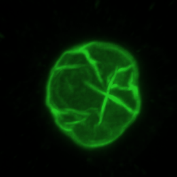

Supplement: Supplementary file 5 — Source data Fig. 3 [file 44319_2026_753_MOESM5_ESM.zip › Figure 3/3P/3P_LaminB1_MG132.tiff]

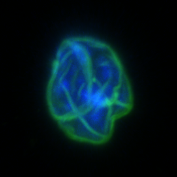

Supplement: Supplementary file 5 — Source data Fig. 3 [file 44319_2026_753_MOESM5_ESM.zip › Figure 3/3P/3P_Merged_BafA1.tiff]

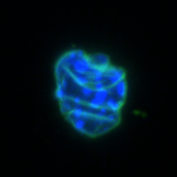

Supplement: Supplementary file 5 — Source data Fig. 3 [file 44319_2026_753_MOESM5_ESM.zip › Figure 3/3P/3P_Merged_DMSO.tiff]

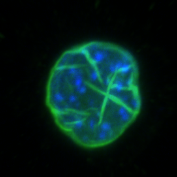

Supplement: Supplementary file 5 — Source data Fig. 3 [file 44319_2026_753_MOESM5_ESM.zip › Figure 3/3P/3P_Merged_MG132.tiff]

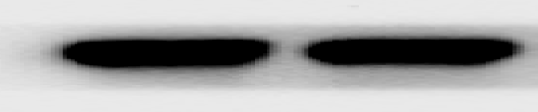

Supplement: Supplementary file 6 — Source data Fig. 4 [file 44319_2026_753_MOESM6_ESM.zip › Figure 4/4B/GAPDH.tif]

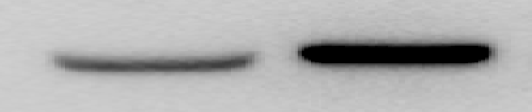

Supplement: Supplementary file 6 — Source data Fig. 4 [file 44319_2026_753_MOESM6_ESM.zip › Figure 4/4B/LaminB1.tif]

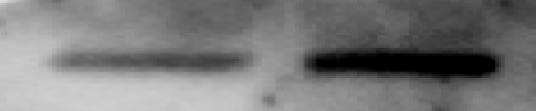

Supplement: Supplementary file 6 — Source data Fig. 4 [file 44319_2026_753_MOESM6_ESM.zip › Figure 4/4B/Midnolin.tif]

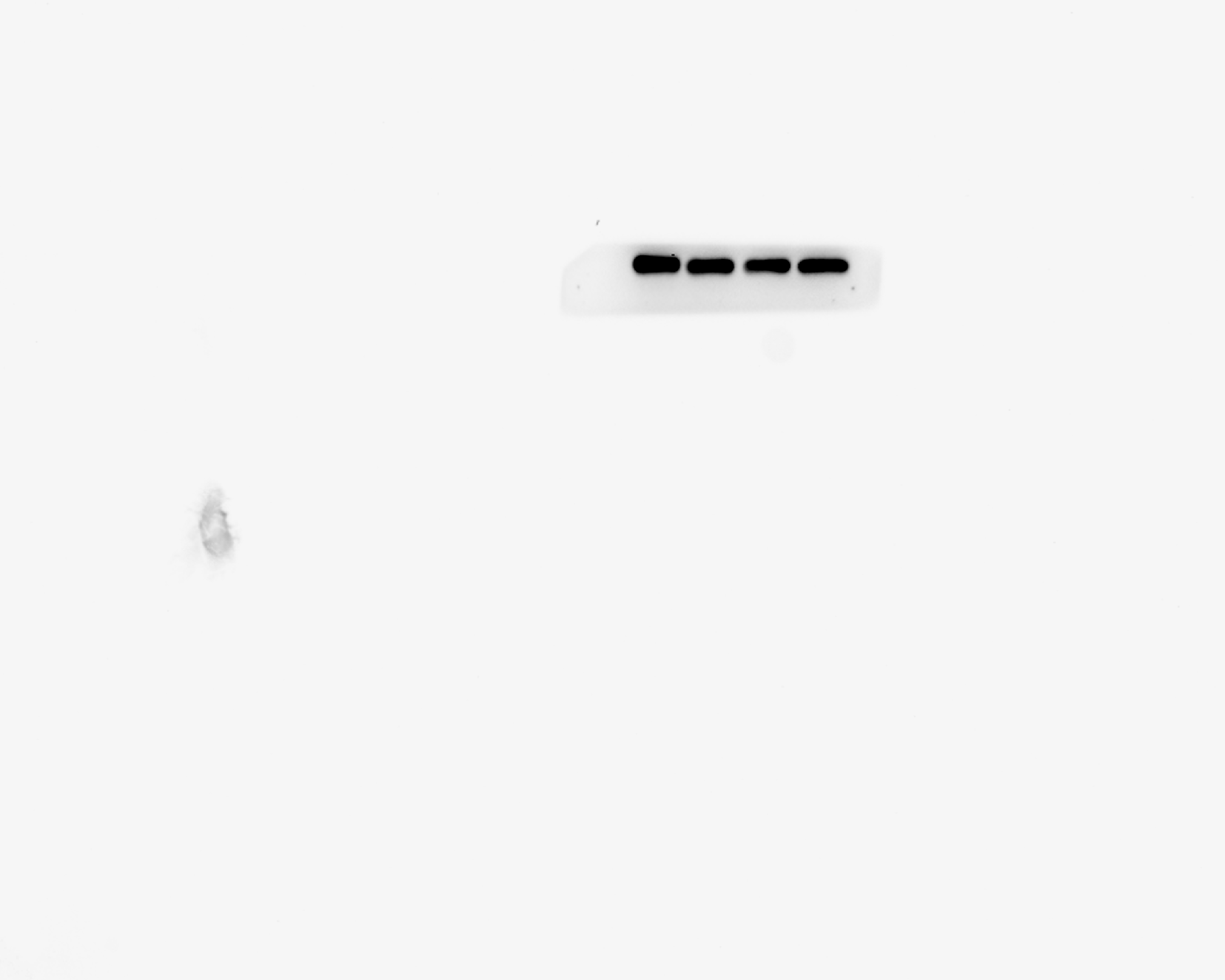

Supplement: Supplementary file 6 — Source data Fig. 4 [file 44319_2026_753_MOESM6_ESM.zip › Figure 4/4D/GAPDH.tif]

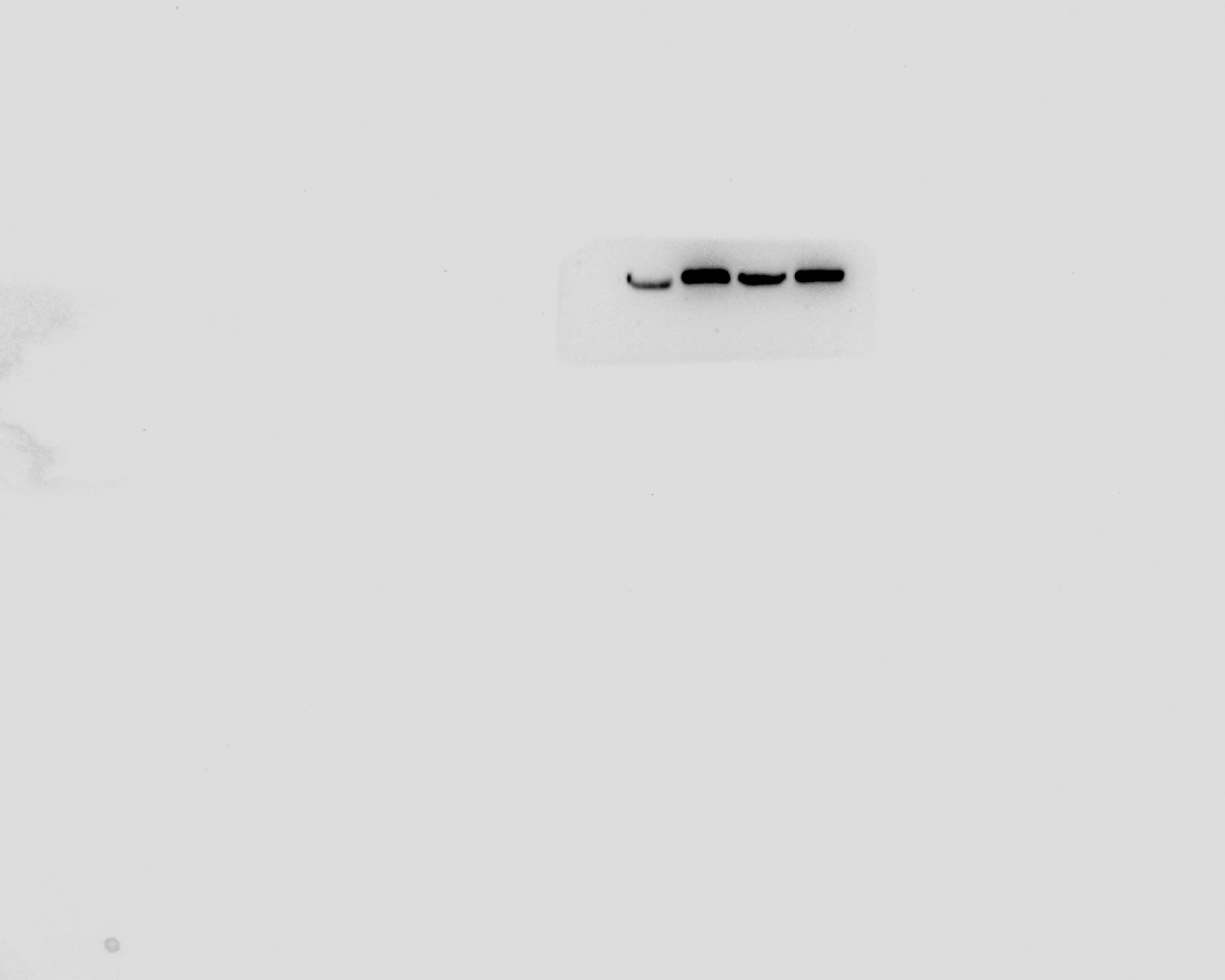

Supplement: Supplementary file 6 — Source data Fig. 4 [file 44319_2026_753_MOESM6_ESM.zip › Figure 4/4D/Lamin B1.tif]

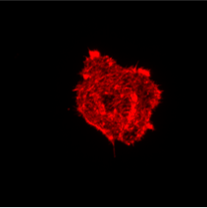

Supplement: Supplementary file 6 — Source data Fig. 4 [file 44319_2026_753_MOESM6_ESM.zip › Figure 4/4F/4F_Factin_0.2kPa_siMidn.tif]

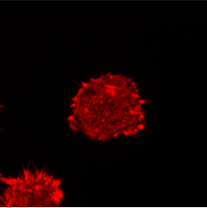

Supplement: Supplementary file 6 — Source data Fig. 4 [file 44319_2026_753_MOESM6_ESM.zip › Figure 4/4F/4F_Factin_0.2kPa_siNC.tif]

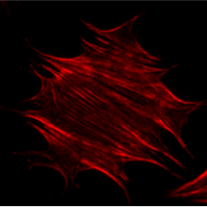

Supplement: Supplementary file 6 — Source data Fig. 4 [file 44319_2026_753_MOESM6_ESM.zip › Figure 4/4F/4F_Factin_10kPa_siMidn.tif]

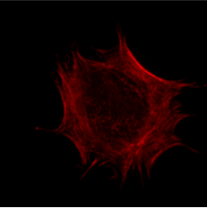

Supplement: Supplementary file 6 — Source data Fig. 4 [file 44319_2026_753_MOESM6_ESM.zip › Figure 4/4F/4F_Factin_10kPa_siNC.tif]

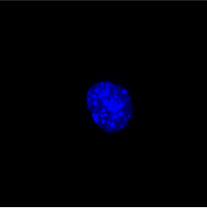

Supplement: Supplementary file 6 — Source data Fig. 4 [file 44319_2026_753_MOESM6_ESM.zip › Figure 4/4F/4F_Hoechst_0.2kPa_siMidn.tif]

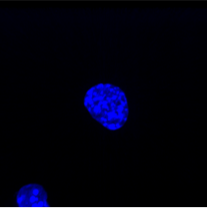

Supplement: Supplementary file 6 — Source data Fig. 4 [file 44319_2026_753_MOESM6_ESM.zip › Figure 4/4F/4F_Hoechst_0.2kPa_siNC.tif]

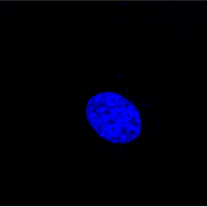

Supplement: Supplementary file 6 — Source data Fig. 4 [file 44319_2026_753_MOESM6_ESM.zip › Figure 4/4F/4F_Hoechst_10kPa_siMidn.tif]

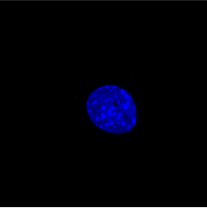

Supplement: Supplementary file 6 — Source data Fig. 4 [file 44319_2026_753_MOESM6_ESM.zip › Figure 4/4F/4F_Hoechst_10kPa_siNC.tif]

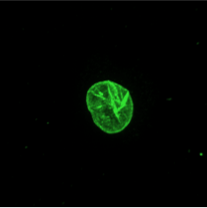

Supplement: Supplementary file 6 — Source data Fig. 4 [file 44319_2026_753_MOESM6_ESM.zip › Figure 4/4F/4F_LaminB1_0.2kPa_siMidn.tif]

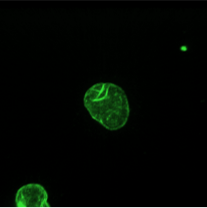

Supplement: Supplementary file 6 — Source data Fig. 4 [file 44319_2026_753_MOESM6_ESM.zip › Figure 4/4F/4F_LaminB1_0.2kPa_siNC.tif]

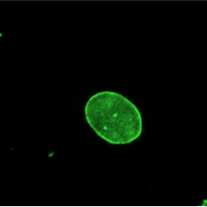

Supplement: Supplementary file 6 — Source data Fig. 4 [file 44319_2026_753_MOESM6_ESM.zip › Figure 4/4F/4F_LaminB1_10kPa_siMidn.tif]

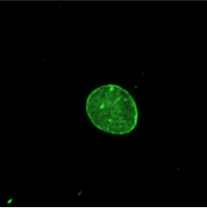

Supplement: Supplementary file 6 — Source data Fig. 4 [file 44319_2026_753_MOESM6_ESM.zip › Figure 4/4F/4F_LaminB1_10kPa_siNC.tif]

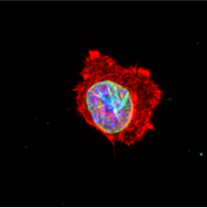

Supplement: Supplementary file 6 — Source data Fig. 4 [file 44319_2026_753_MOESM6_ESM.zip › Figure 4/4F/4F_Merged_0.2kPa_siMidn.tif]

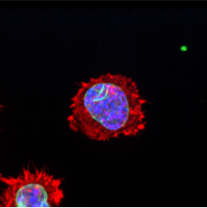

Supplement: Supplementary file 6 — Source data Fig. 4 [file 44319_2026_753_MOESM6_ESM.zip › Figure 4/4F/4F_Merged_0.2kPa_siNC.tif]

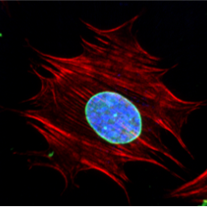

Supplement: Supplementary file 6 — Source data Fig. 4 [file 44319_2026_753_MOESM6_ESM.zip › Figure 4/4F/4F_Merged_10kPa_siMidn.tif]

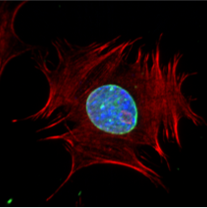

Supplement: Supplementary file 6 — Source data Fig. 4 [file 44319_2026_753_MOESM6_ESM.zip › Figure 4/4F/4F_Merged_10kPa_siNC.tif]

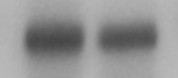

Supplement: Supplementary file 6 — Source data Fig. 4 [file 44319_2026_753_MOESM6_ESM.zip › Figure 4/4I/4I_Input_GFP.tif]

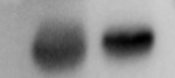

Supplement: Supplementary file 6 — Source data Fig. 4 [file 44319_2026_753_MOESM6_ESM.zip › Figure 4/4I/4I_Input_LaminB1.tif]

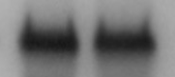

Supplement: Supplementary file 6 — Source data Fig. 4 [file 44319_2026_753_MOESM6_ESM.zip › Figure 4/4I/4I_IP_GFP.tif]

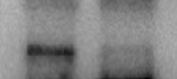

Supplement: Supplementary file 6 — Source data Fig. 4 [file 44319_2026_753_MOESM6_ESM.zip › Figure 4/4I/4I_IP_LaminB1.tif]

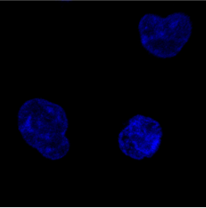

Supplement: Supplementary file 6 — Source data Fig. 4 [file 44319_2026_753_MOESM6_ESM.zip › Figure 4/4K/4K_DAPI_0.2kPa.tif]

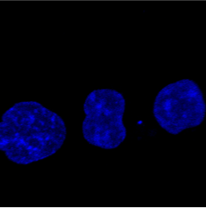

Supplement: Supplementary file 6 — Source data Fig. 4 [file 44319_2026_753_MOESM6_ESM.zip › Figure 4/4K/4K_DAPI_10kPa.tif]

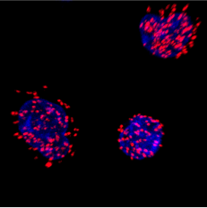

Supplement: Supplementary file 6 — Source data Fig. 4 [file 44319_2026_753_MOESM6_ESM.zip › Figure 4/4K/4K_Merged_0.2kPa.tif]

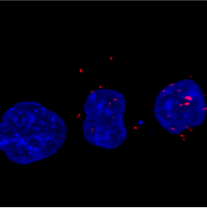

Supplement: Supplementary file 6 — Source data Fig. 4 [file 44319_2026_753_MOESM6_ESM.zip › Figure 4/4K/4K_Merged_10kPa.tif]

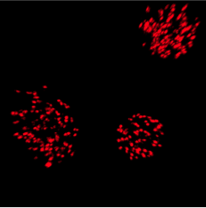

Supplement: Supplementary file 6 — Source data Fig. 4 [file 44319_2026_753_MOESM6_ESM.zip › Figure 4/4K/4K_PLA_0.2kPa.tif]

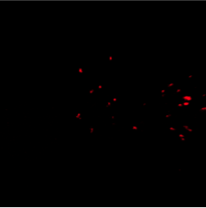

Supplement: Supplementary file 6 — Source data Fig. 4 [file 44319_2026_753_MOESM6_ESM.zip › Figure 4/4K/4K_PLA_10kPa.tif]

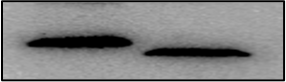

Supplement: Supplementary file 6 — Source data Fig. 4 [file 44319_2026_753_MOESM6_ESM.zip › Figure 4/4O/INPUT_FLAG.tif]

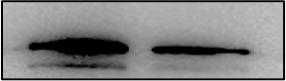

Supplement: Supplementary file 6 — Source data Fig. 4 [file 44319_2026_753_MOESM6_ESM.zip › Figure 4/4O/INPUT_GFP.tif]

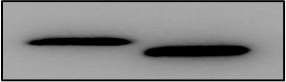

Supplement: Supplementary file 6 — Source data Fig. 4 [file 44319_2026_753_MOESM6_ESM.zip › Figure 4/4O/IP_FLAG.tif]

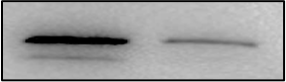

Supplement: Supplementary file 6 — Source data Fig. 4 [file 44319_2026_753_MOESM6_ESM.zip › Figure 4/4O/IP_GFP.tif]
