# Supplementary material for: Matrix stiffness induces midnolin-dependent lamin B1 degradation to control myoblast differentiation
Source: EMBO Rep. 2026 Mar 31;27(9):2297–318. doi: 10.1038/s44319-026-00753-0 (PMC13172543; doi:10.1038/s44319-026-00753-0)
Supplement: Supplementary file 8 — Expanded View Figures [file 44319_2026_753_MOESM8_ESM.pdf]

Expanded View Figures

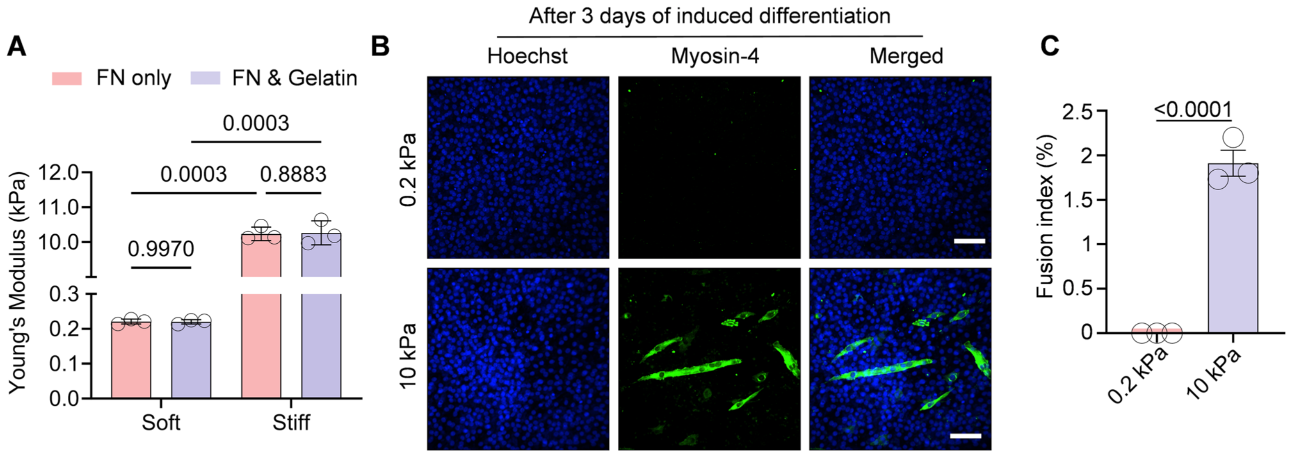

Figure EV1. Young's modulus of PAA gels and early myoblast differentiation on different matrix.

(**A**) Young's modulus (*E*, kPa) of PAA hydrogels coated with FN only versus FN combined with Gelatin ($n = 3$ biological replicates. Data are presented as the mean ± SD). (**B**) Representative images of differentiated C2C12 cells on FN and gelatin-coated PAA matrices after 3 days of 2% horse serum induction. scale bar, 100 µm. (**C**) Quantification data of the fusion index in (**B**). $n = 3$ biological replicates, Data are presented as the mean ± SD. Two-tailed Student's *t* test. $P = 9.96 \times 10^{-5}$.

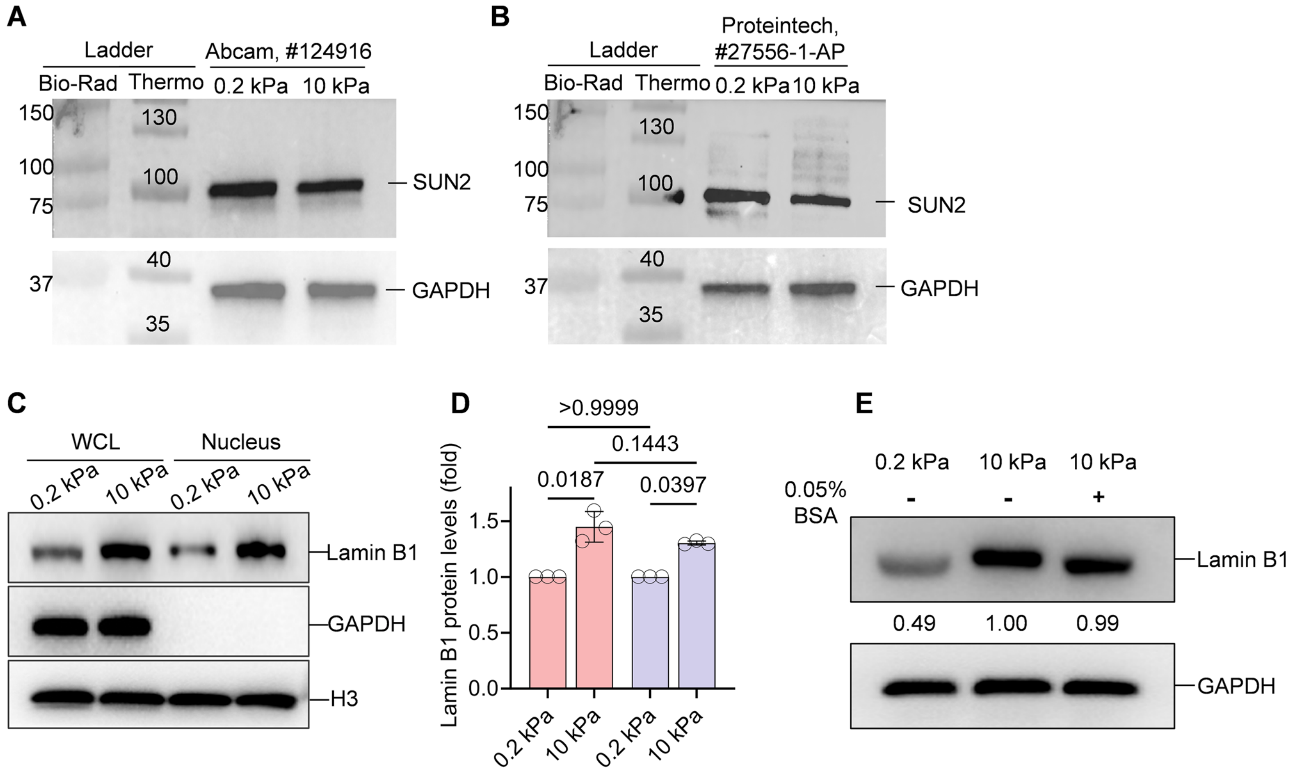

Figure EV2. Validation of the molecular weight of SUN2 band and the band shape of Lamin B1.

(**A**) SUN2 was detected by primary antibody from Abcam, which was used in this study. The band was aligned with two proteins ladder (Thermo Scientific ladder, Cat#26616 and Bio-Rad ladder, Cat# 1610374) and displayed molecular weight differently. (**B**) Same as (**A**), but detected by a different primary antibody from Proteintech. (**C**) Lamin B1 protein levels and band shapes were examined with whole cell lysates (WCL) or nuclear fractions (Nucleus) from FN-coated 0.2 kPa or 10 kPa gels. (**D**) Statistical analysis of lamin B1 protein levels among the groups from (**C**). $n = 3$ biological replicates. Data are presented as the mean ± SD. Tukey's multiple comparisons test. (**E**) Lamin B1 protein levels and band shapes were examined with or without BSA added. Protein samples were whole cell lysates from soft gels or stiff gels coated with FN.

© The Author(s)

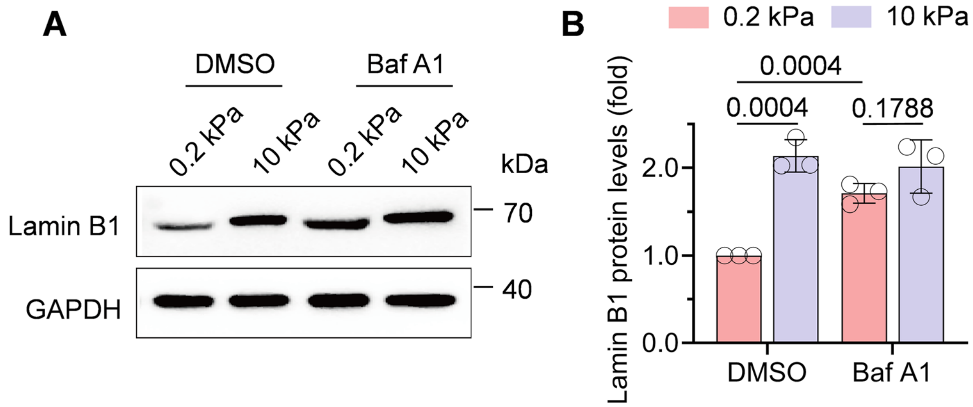

Figure EV3. Bafilomycin A1 treatment on matrix.

(**A**, **B**) Western blot analysis of lamin B1 protein in C2C12 seeded onto FN and gelatin-coated PAA matrices for 30 min in the presence of degradation inhibitor (Baf A1, 100 μM). $n = 3$ biological replicates, Data are presented as the mean ± SD. Two-tailed Student's t test.

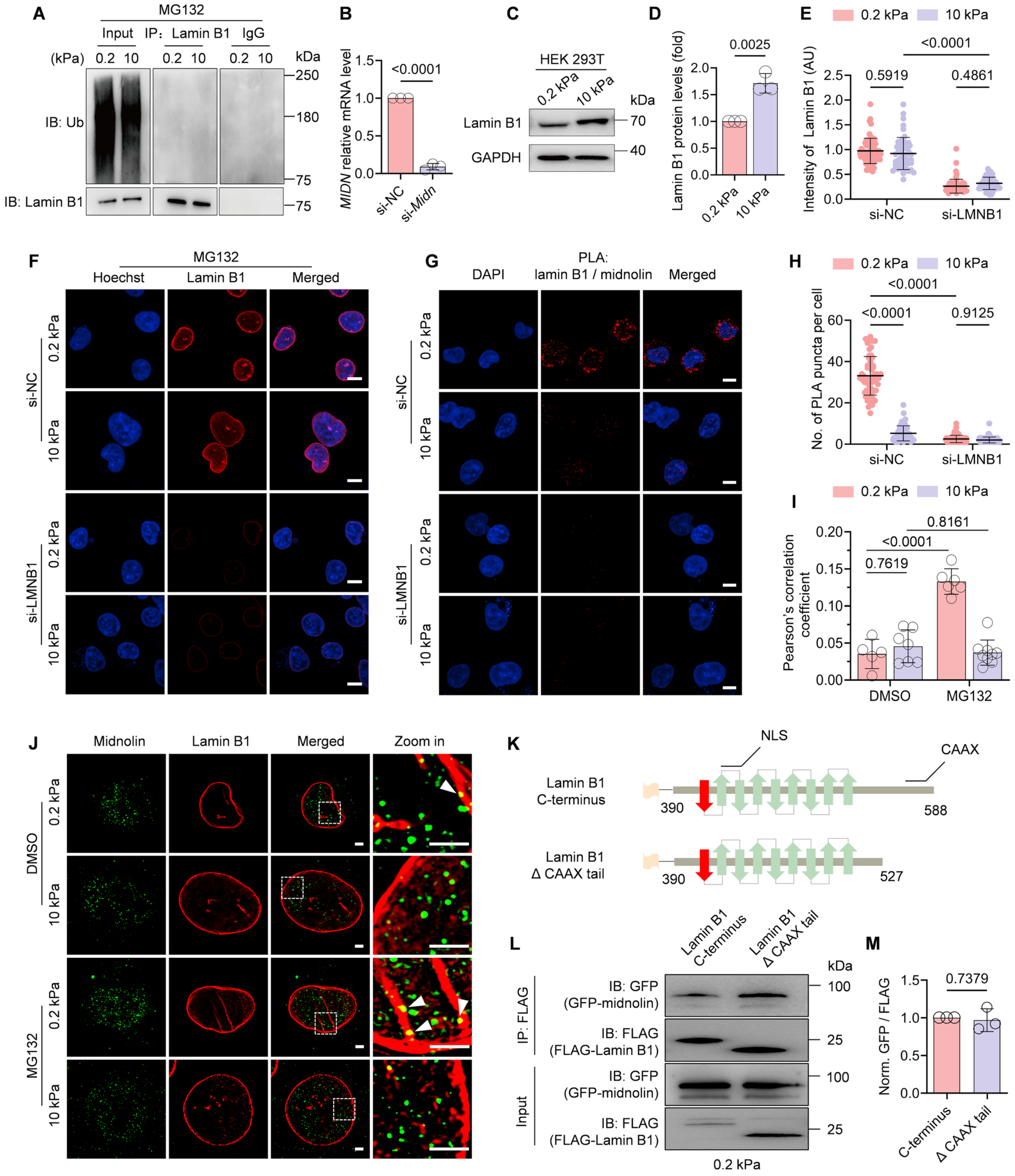
A
MG132
Input IP: Lamin B1 IgG
(kPa) 0.2 10 0.2 10 0.2 10
kDa
250
180
75
75
IB: Ub
IB: Lamin B1
B
MIDN relative mRNA level
<0.0001
si-NC
si-Midn
C
HEK 293T
0.2 kPa
10 kPa
kDa
Lamin B1
GAPDH
70
40
D
Lamin B1 protein levels (fold)
0.0025
0.2 kPa
10 kPa
E
0.2 kPa 10 kPa
Intensity of Lamin B1 (AU)
<0.0001
0.5919
0.4861
si-NC
si-LMNB1
F
MG132
Hoechst
Lamin B1
Merged
si-NC
si-LMNB1
0.2 kPa
10 kPa
G
PLA:
DAPI
lamin B1 / midnolin
Merged
H
No. of PLA puncta per cell
<0.0001
<0.0001
0.9125
I
Pearson's correlation coefficient
0.8161
<0.0001
0.7619
DMSO
MG132
J
Midnolin
Lamin B1
Merged
Zoom in
DMSO
MG132
K
NLS
CAAX
Lamin B1 C-terminus
390
588
Lamin B1 Δ CAAX tail
390
527
L
Lamin B1 C-terminus
Lamin B1 Δ CAAX tail
kDa
IP: FLAG
Input
IB: GFP (GFP-midnolin)
IB: FLAG (FLAG-Lamin B1)
100
25
0.2 kPa
M
Norm. GFP / FLAG
0.7379
C-terminus
Δ CAAX tail

Figure EV4. Validation of the specificity of the interaction between Lamin B1 and midnolin.

(**A**) The ubiquitination level of lamin B1 in C2C12 with MG132. Cells were pre-treated with MG132 for 6 h and seeded on FN and gelatin-coated PAA matrices for 0.5 h, the cells were then harvested with IP lysis buffer. IP assay was performed with anti-ubiquitin. (**B**) qRT-PCR to detect the expression of *Midn* transduced with si-*Midn*. $n = 3$ biological replicates. Data are presented as the mean ± SD. Two-tailed Student's *t* test was used for statistical analysis. $P = 3.04 \times 10^{-6}$. (**C**, **D**) Western blot analysis of lamin B1 proteins in HEK293T cells seeded onto FN-coated PAA matrices after 0.5 h. $n = 3$ biological replicates. (**E**, **F**) Quantification data of lamin B1 intensity and corresponding representative images from negative control scramble siRNA (si-NC) and Lamin B1 knockdown (si-*LMNB1*) HEK293T cells with MG132 treatment on FN-coated PAA matrices for 3 h. Scale bars, 10 μm. (Tukey's multiple comparisons test. Data were presented as the mean ± SD. >50 cells for each condition). In (**E**), *P* values are 0.5919, 2×10^{-10}, and 0.4861, respectively. (**G**) Representative images of PLA to detect the interaction between lamin B1 and GFP-midnolin in HEK293T cells with si-NC or si-LMNB1 on FN-coated PAA matrices for 3 h with MG132 treatment. Scale bar: 10 μm. (**H**) Quantification of PLA signals from experiments as in (**G**) (Tukey's multiple comparisons test. Data were presented as the mean ± SD. >50 cells for each condition). In (**E**), *P* values are 1×10^{-10}, 1×10^{-10}, and 0.9125, respectively. (**I**, **J**) Representative SIM images of midnolin (green) and lamin B1 (red) in C2C12 cells on FN and gelatin-coated PAA matrices with DMSO or MG132 treatment for 0.5 h. The yellow dots are the colocalization of midnolin and lamin B1 (white arrows). Scale bars, 2 μm. Pearson's correlation coefficient was calculated for lamin B1 and midnolin based on the imaging results in (**J**). $n > 5$ cells for each condition. In (**I**), *P* values are 0.7619, 9.26×10^{-8}, and 0.8161, respectively. (**K**) Schematics of lamin B1 Ig-like domain and CAAX motif truncation. (**L**) HEK293T cells were transfected with Lamin B1 Ig-like domain or CAAX motif truncation and cultured on FN-coated 0.2 kPa gels for 3 h. (**M**) Normalized midnolin/lamin B1 intensity in (**J**). $n = 3$ biological replicates. Data (**B**, **D**, **I**, **M**) are the mean ± SD, with two-tailed Student's *t* test.

© The Author(s)

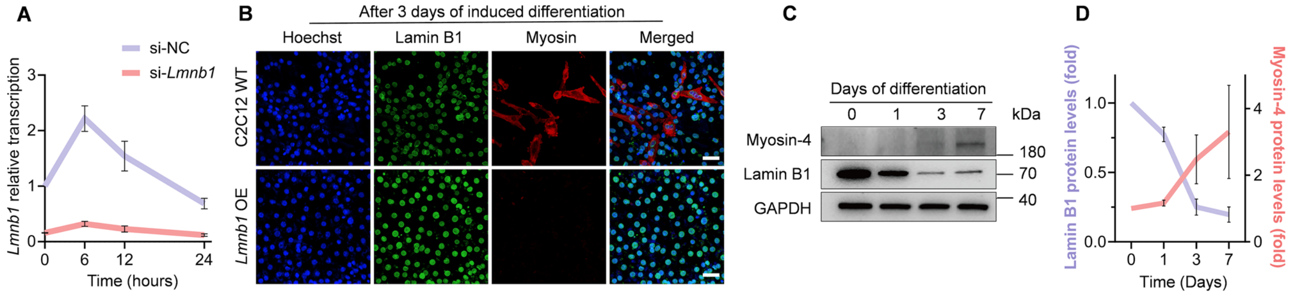

Figure EV5. Myoblast differentiation requires moderate lamin B1 protein levels.

(**A**) qPCR to examine the expression of *Lmnb1* during 24 h differentiation with scramble siRNA (si-NC) and si-*Lmnb1*. $n = 3$ biological replicates. Data are presented as the mean ± SD. (**B**) Representative images of WT and *Lmnb1* overexpression C2C12 cells on Petri-dishes with 2% horse serum induced differentiation for 3 days. Scale bar, 50 µm. (**C**, **D**) Western blot analysis of lamin B1 protein and Myosin-4 protein in C2C12 during 2% horse serum induced differentiation and corresponding intensity quantification data. $n = 3$ biological replicates. Data are presented as the mean ± SD.

© The Author(s)
